# Analytical ultracentrifugation in saliva research: Impact of green tea astringency and its significance on the *in-vivo* aroma release

**DOI:** 10.1038/s41598-018-31625-w

**Published:** 2018-09-06

**Authors:** Vlad Dinu, Chujiao Liu, Joseph Ali, Charfedinne Ayed, Pavel Gershkovich, Gary G. Adams, Stephen E. Harding, Ian D. Fisk

**Affiliations:** 1 Division of Food Sciences, School of Biosciences, University of Nottingham, Sutton Bonington Campus, Nottingham, Leicestershire UK; 2 National Centre of Macromolecular Hydrodynamics, Division of Food Sciences, School of Biosciences, University of Nottingham, Sutton Bonington Campus, Nottingham, Leicestershire UK; 3 School of Pharmacy, University of Nottingham, Nottingham, UK; 4 School of Health Sciences, Faculty of Medicine and Health Sciences, Queen’s Medical Centre, Clifton Boulevard, Nottingham, UK

## Abstract

Current saliva testing methods rely on cutting edge yet expensive techniques for the detection and analysis of genetic material, proteins and biomarkers for clinical use. However, these techniques are limited in scope and often cannot be used with complex food materials. We propose an efficient *ex*-*vivo* tool for evaluating biologically relevant interactions between food components and human saliva using sedimentation velocity analytical ultracentrifugation (SV-AUC). We evaluated macromolecular content from “unstimulated” (US) and “stimulated” (SS) samples pooled from 5 healthy volunteers. Over 90% of total saliva protein consisted of α-amylase and mucin, and up to 10% was secretory immunoglobulin A (SIgA). It was shown that α-amylase concentration increased upon parafilm stimulation, which lead to a decrease in the viscosity of saliva. Then, we used a simple food system (green tea) to evaluate changes in the salivary protein content caused by green tea polyphenols. It was found that aroma release from green tea is highly influenced by interactions between α-amylase and polyphenol epigallocatechin 3-gallate (EGCG). This interaction was found to increase the viscosity of the salivary bulk, suggested to contribute to astringency, and increased the concentrations of β-ionone, benzaldehyde and isovaleraldehyde (*P* < 0.01), suggested to play a significant role in the characteristic flavour of green tea.

## Introduction

Astringency is a complex phenomenon in which some food or drug compounds cause a puckering (or drying) feeling of the oral epithelium^1^. It is considered to be a tactile sensation caused by a loss of lubrication and increased friction in the salivary film^2,3^. Recently, there has been an increasing interest into trying to understand the development of astringency. However, in understanding its complexity, it is essential to understand normal oral physiology conditions, which are maintained by a thin hydrated layer of saliva. Whole saliva is secreted by three major salivary glands: parotid, sublingual and submandibular glands. Over 99% is made up of water, while the remaining 1% consists of a variety of salts and proteins, responsible for the physiochemical properties of saliva, i.e. pH, buffering capacity, viscosity, lubrication^4,5^, starch digestion^6,7^ and other functions, such as contribution to speech, taste perception and tooth remineralisation^8^. The most abundant salivary proteins/glycoproteins are salivary mucins MUC5B and MUC7, secretory immunoglobulin A (SIgA), salivary α-amylase, carbonic anhydrase, histatins, statherins and proline rich proteins^9^.

“Unstimulated” saliva (US) represents a steady and continuous secretion forming the salivary film on the oral epithelium. Under stimulation (SS), there is a marked increase in the concentration of salivary salts and certain proteins, which elicit a marked change in the physiochemical characteristics of unstimulated saliva. For instance, the contribution of parotid saliva increases from 30% to 70% during stimulation^10,11^.

Mucins are secreted by the submandibular and sublingual glands and play a key role in lubricating the oral cavity. MUC5B and MUC7 have been amongst the first oral mucins to be characterized^12^. Electrophoresis experiments found that MUC5B contains high molecular weight gelling fractions (>1 MDa), while MUC7 consists of a much smaller, soluble fraction of (<300 kDa)^12^. The structures of these two types of salivary mucins differ considerably. On one hand, MUC5B contains ~5000 amino-acid residues with an overall tri-block structure, similar to early described ‘windmill’ models^13^. It was found that the N terminus of MUC5B contains a number of von Willebrand factor (haemostatic glycoprotein) type D-domains (vWF), which contain cysteine and other charged amino acid residues. On the other hand, MUC7 mucins were not found to contain vWF regions^14^, rendering MUC7 mucins incapable to gel.

Low molecular weight proline rich proteins (PRPs) and salivary α-amylase are two other major proteins, produced by the parotid gland^12,15^. Previous work has shown that enzymes, mucins and PRPs can be precipitated by polyphenols, which is believed to lead to the perception of astringency. Due to the high polyphenol content in tea and its antioxidative functions, regular intake of polyphenols and tannins from tea has long been associated with a healthier lifestyle^16^. Studies have shown that green tea polyphenol extract is composed of epicatechin, epicatechin gallate and epigallocatechin gallate (EGCG)^17^. Over 50% of green tea polyphenols are EGCG, previously reported to have various biological effects, including the inhibition of digestive enzymes, such as α-amylase. Therefore, in addition to its impact on taste and antioxidant capacity, its use has also been considered for the treatment of diabetes and obesity^18,19^.

The present study describes an efficient tool for characterising the macromolecular content in “stimulated” (SS) and “unstimulated” (US) saliva using sedimentation velocity-analytical ultracentrifugation (SV-AUC). With regard to macromolecular analysis of human saliva, Analytical Ultracentrifugation offers numerous advantages over other size determination techniques, such as size exclusion chromatography, or gel electrophoresis^20^. As such, it requires no matrix for the separation of macromolecules, therefore it is assumed that there is no loss of sample components during analysis. Preparations can be analysed in their native state, at physiological conditions and concentrations. Although saliva contains a large number of macromolecular species, their relative concentration in human saliva is negligible with regard to complex food applications. Consequently, we focus on the most abundant macromolecules in submandibular/sublingual saliva, i.e. α-amylase and mucin. The study is designed to assess changes in their concentration during food consumption. Using the powerful, matrix free technique of SV-AUC, we have shown that interactions between green tea epigallocatechin 3-gallate (EGCG) and salivary α-amylase promote the release of specific volatile aroma compounds. These *in-vivo* interactions may lead to further understanding the process of aroma delivery from green tea and help towards improving the flavour profile of tea based beverages.

## Results and Discussion

### Sedimentation fingerprinting of whole human saliva

**Figure 1:**
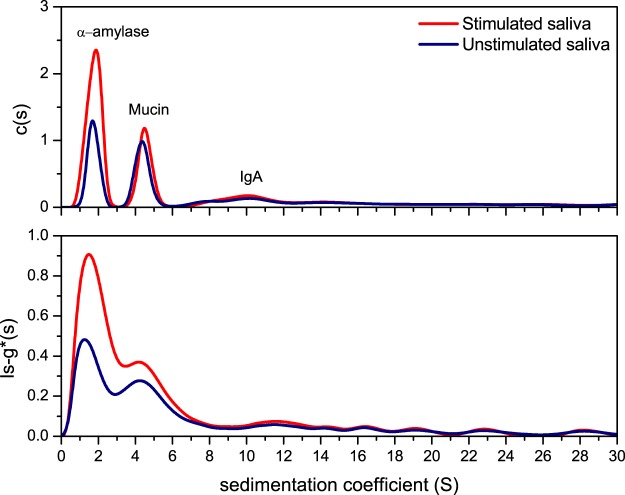
Sedimentation velocity, ls-g*(s) and c(s) analysis for pooled saliva. The plots show the sedimentation coefficient distributions for unstimulated (US) and stimulated (SS) samples. Saliva fingerprinting is based on the sedimentation coefficient distribution, directly derived by fitting the Lamm equation in SEDFIT^20,25,46^. The plots shows typical sedimentation coefficient profiles for the sedimentation species present in saliva analysed by two methods, c(s) (top) and ls-g*(s) (bottom). Peaks are identified based on their molecular weight (directly related to the sedimentation coefficient) and their relative concentration. Estimations have been confirmed with individual runs for porcine α-amylase and bovine submaxillary mucin. The US and SS distributions are shown in blue and red respectively. Rotor speed: 40 000 rpm (120 000 g), 20.0 °C.

Sedimentation coefficient distributions for human US and SS saliva were analysed by two methods, ls-g*(s) and the diffusion deconvoluted model c(s)^21^. The experiment confirmed the presence of three major macromolecular components (Fig. 1). The values of the weighted averaged sedimentation coefficients (s_20,w_) have been paralleled by experiments using standard solutions of porcine α-amylase (1.5S) and bovine submaxillary mucin (4S), while the sedimentation coefficient of SIgA (11S) is commensurate with previous established reports^22,23^. Total protein concentration was approximated using a differential refractometer using an average *dn/dc* of 0.18 mL/g^24^. Each individual fraction was then calculated in terms of the proportion of its loading concentration (fringe units) using the integration tool in SEDFIT^25^ and are presented in Table 1. The relative viscosities (t_s_/t_0_) of SS and US samples were calculated against t_0_ for 0.1 M PBS, pH 6.8^26^.

**Table 1:** Relative viscosity and protein concentrations for unstimulated (US) and stimulated (SS) human saliva. Data shown as mean +/− SD, n = 3.

Pooled saliva samples	Total protein concentration (mg/mL)	Relative viscosity	Peak 1 *α-Amylase* (mg/mL)	Peak 2 *Mucin* (mg/mL)	Peak 3 *IgA* (mg/mL)	Other (mg/mL)
*US*	2.36(±0.09)	1.045	0.85(±0.03)	0.94(±0.05)	0.28(±0.02)	0.28(±0.03)
*SS*	3.41(±0.15)	1.035	1.91(±0.05)	0.95(±0.05)	0.28(±0.05)	0.19(±0.07)

**Figure 2:**
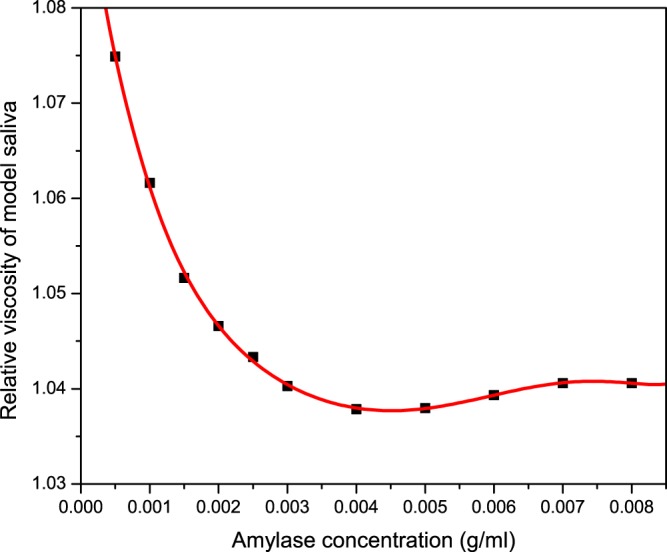
Relative viscosity analysis showing the quantitative effect of α-amylase addition to bovine submaxillary mucin (1.0 mg/mL) in 0.1 M phosphate buffer saline. The polynomial fit is based on 11 datapoints derived from separate amylase/mucin solutions. Each solultion contaed a constant concentration of submaxillary mucin of 1 mg/mL, while procine α-amylase concentration ranged from 0.5 mg/mL to 8.0 mg/mL, to mimic the physiological range in saliva. Each solution was injected into the Ostwald capillary and the drop times were recorded and used to derive their relative viscosities.

The SS sample was found to contain a higher overall protein concentration (Table 1). From the sedimentation distribution trace, it is suggested that a higher α-amylase concentration of sedimentation coefficient (1.5S), is responsible for the higher protein content in SS. The mucin concentration was shown to remain constant at ~0.95 mg/mL. In our study, it is highly likely that the mucin peak corresponds to MUC7 type, since MUC5B types are known to gel and have most likely been removed during preparative sedimentation steps, due to their very large molecular size (2–50 MDa). Consequently, the viscosity results obtained in our study may be affected. However, results are in agreement with similar studies showing a lower viscosity for SS, and this is thought to be attributed to the higher proportion of α-amylase, and small proline rich proteins (PRPs), which have very low intrinsic viscosities, hence lowering the relative viscosity of the saliva^8,10^. This has later been confirmed by capillary viscometry (Fig. 2).

Results in Fig. 2 show that there is a decrease in the relative viscosity of our model saliva upon gradual addition of α-amylase. The concentration of submaxillary mucin was kept constant at 1 mg/mL while the concentration of porcine α-amylase increased from 0.5 mg/mL to 8.0 mg/mL, to represent physiological ranges of α-amylase in human saliva^15^. The average concentration in human saliva was previously estimated at (2.64 ± 1.8) mg/ml^15^, which fits within the viscosity range for US and SS, as shown in Table 1.

Once a reliable sedimentation coefficient fingerprint for human saliva has been established, the next step was to characterize the interactions between saliva and green tea, and identify which of the salivary proteins are affected by tea polyphenols.

### Saliva interactions with green tea

**Figure 3:**
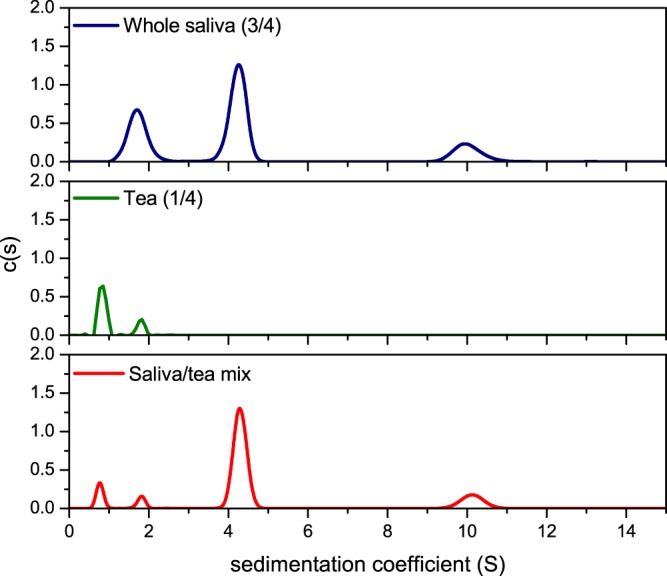
Sedimentation velocity, c(s) analysis showing the sedimentation coefficient distributions for diluted whole US saliva (blue line), diluted green tea (green) and their interaction complex (red). The plot shows combined sedimentation coefficient distributions for individual US saliva (blue) and diluted green tea (green) as well as their combination. A 25% dilution step was required to maintain a constant concentration of saliva throughout the analysis, and to allow the mixing with tea. A complete loss in the amylase peak is indicative of an interaction between α-amylase and green tea constituents. Rotor speed: 40 000 rpm (120 000 g), 20.0 °C.

Figure 3 shows the mixture between green tea and human saliva analysed by the c(s) method from SEDFIT, with distributions recorded using interference optics. While the saliva sample shows the presence of the three characteristic peaks, the green tea sample (diluted) has resolved the presence of two macromolecular components, at ~1S and ~2S. In the presence of green tea, the distribution of sedimentation coefficients in saliva show a loss of the amylase peak. This is indicative of a direct interaction between the green tea components and human α-amylase. By contrast, there is no significant change in the distribution of mucin and SIgA peaks as compared to the controls.

**Figure 4:**
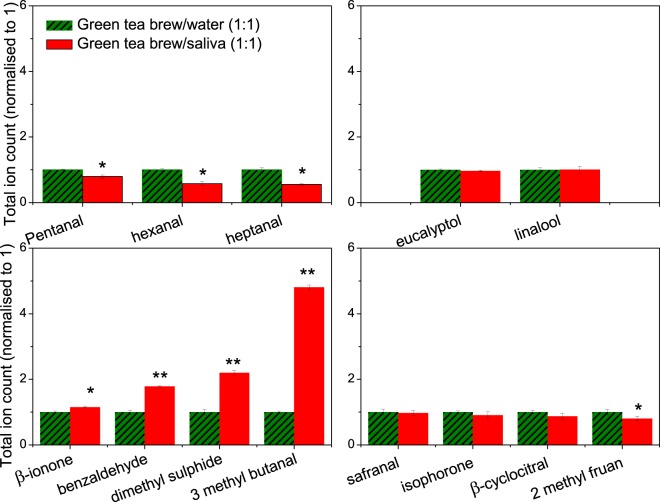
Effect of green tea on the release of aroma compounds from whole saliva. The dashed green columns express the concentration of green tea aroma compounds, normalised to one unit, while the red columns represent the headspace concentration for the saliva-tea mixture relative to that of the green tea. The data was divided based on the effect of mixing. The comparison is made by Tukey’s post hoc test to calculate the *P*-values (*P* < 0.01**, *P* < 0.05*). The data shown as mean +/− SD, n = 3.

To study the effect of this interaction on aroma development, a GC-MS experiment has been performed to analyse changes in the partitioning of volatile compounds upon mixing with saliva (Fig. 4). A1:1 volume ratio has been considered, corresponding to 5 mL of saliva and 5 mL of tea. The green tea control has also been diluted 1:1 with water, to account for any changes due to dilution.

**Figure 5:**
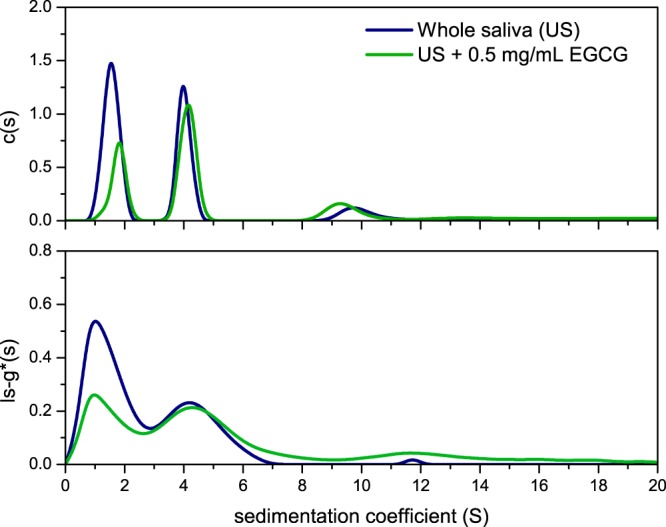
Sedimentation velocity, ls-g*s(s) and c(s) analyses showing the sedimentation coefficient distributions for whole US saliva (blue line) and what happens upon the addition of epigallocatechin gallate (EGCG) (green line). When 0.5 mg/mL EGCG was added to US saliva, the amylase concentration was reduced. This confirmed the interaction between EGCG and human salivary α-amylase. The assay was confirmed by using porcine α-amylase as a standard (unpublished). Rotor speed: 40 000 rpm (120 000 g), 20.0 °C.

Of the aroma compounds identified in green tea, 13 compounds were characterised by GC-MS. Differences were considered significant at *P* < 0.05 (*) and *P* < 0.01 (**). The presence of salivary proteins had significant effect on the partitioning of a number of volatile compounds, although terpene alcohols such as eucalyptol and linalool showed no significant change in their headspace concentration (*P* > 0.05). A significant decrease in the relative abundance in the headspace was observed for the alkyl aldehydes (*P* < 0.05), such as pentanal, hexanal and heptanal. These effects have been attributed to Schiff base adduct formation between the N-terminal amino groups of α-amylase and the carbonyl group of the aroma compounds^27^. A sharp increase in the relative abundance in the headspace was detected for β-ionone, benzaldehyde, isovaleraldehyde and dimethyl sulphide (*P* < 0.01). Dimethyl sulphide has been reported to be formed during heating of tea in water^28^. Other compounds are known to be formed either by oxidative deamination of amino acids, or by carotenoid oxidation^29^. Previous reports have shown that the oxidation of phenylalanine, valine and leucine by tea quinones can produce characteristic odours such as isovaleraldehyde (3, methyl butanal) and isobutanal. Sanderson *et al*.^30^, have demonstrated that quinone oxidation of β-carotene is converted to β-ionone, while other compounds including benzaldehyde, have been shown to be formed by the hydrolysis of glycosidic bound volatiles^31^. This is suggested to arise from the action of α-amylase, which itself is a glycoside hydrolase acting on α-glucoside bonds. Yilmazer-Musa *et al*.^32^, have found that catechin 3-gallates are potent inhibitors of α-glucosides, but even more potent inhibitors of α-amylase activity. As a result, it is suggested that catechin 3-gallates, abundant in green tea, promote the release of these volatile compounds as a result of their affinity to bind α-amylase. As a result, a SV-AUC experiment was carried out to confirm the interaction (Fig. 5).

The experiment was performed using whole saliva (US), containing ~1.0 mg/mL α-amylase, and by adding 0.5 mg/mL epigallocatechin gallate (EGCG) (Fig. 5). The data, analysed by ls-g*(s) and c(s) methods, confirmed the interaction between EGCG and amylase as indicated by the reduction in the concentration of the 1.5S component, α-amylase. In an analogous manner, we validated the experiment by investigating the effect of EGCG on our model saliva, based on 1 mg/mL bovine submaxillary mucin and 1 mg/mL porcine pancreatic α-amylase (Supplementary Fig. 1). Based on the area under the curve calculations, it was found that the concentration of α-amylase has decreased by 47% upon EGCG addition, commensurate with our results from the real system (Fig. 5).

**Figure 6:**
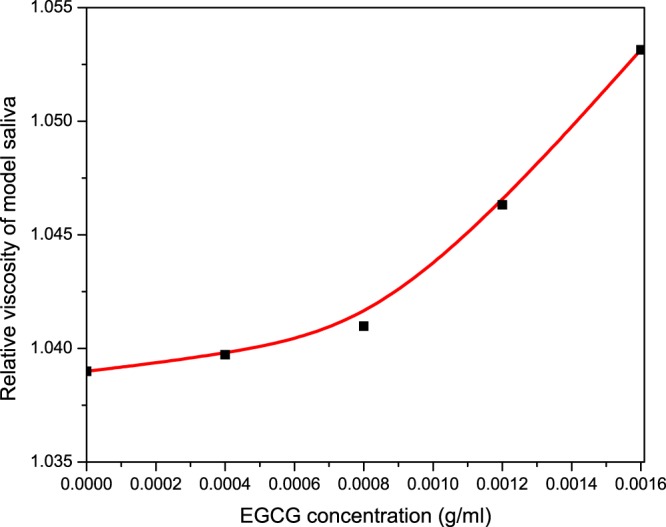
Relative viscosity analysis showing the quantitative effect of EGCG addition to model saliva in 0.1 M phosphate buffer saline, pH 6.8. This assay is based on five datapoints derived from separate solutions. Each solution was made with a constant concentration of amylase (1 mg/mL) and mucin (1 mg/mL), correspoding to our model saliva. Relative viscosity of model saliva is shown at 0.0 mg/mL EGCG concentration. Increasing concentrations of EGCG were added to study its effect on the relative viscosity of our model saliva.

We further examined the impact of this green tea flavonoid on the viscosity of saliva. Our results show that there is a dose dependent increase on the relative viscosity of our model saliva upon EGCG addition (Fig. 6). Yet again, this is thought to be related to the lowering of amylase concentration by EGCG, but also due to the increased friction resulting from protein precipitation. An argument could be made to say that the viscosity data is based on the assumption that porcine pancreatic α-amylase has the same hydrodynamic properties as human salivary α-amylase. Nevertheless, viscosity effects are evident from this investigation and may also play a role in flavour perception through cross-modal perception interactions.

### General remarks

There are limited published reports on the hydrodynamic properties and concentrations of mucin, α-amylase and secretory IgA in saliva, partly due to different collection protocols or different quantification techniques. Although it is difficult to compare reported values, the present results are in agreement with other findings for mucin^33^, α-amylase^15^ and SIgA^22,23^. The viscosity values obtained by other research groups vary, depending on the type of instrumentation and post collection protocols. In our study, saliva has been centrifuged to remove excess aggregates, i.e. MUC5B type mucins, bacteria and dead cells, thereby providing a relatively clean reaction medium. As a result, this may interefere with our hydrodynamic analysis. However, lower α-amylase results for our stimulated samples (SS) are consistent with the majority of findings^5,7^, although conflicting results are identified depending on the nature of the stimulus^34^. In the current study this has been agreed to be due to the increased output of the parotid gland, during parafilm stimulation. Our approach has shown that by increasing the amylase concentration, the relative viscosity of saliva is reduced (Fig. 2).

Mucins in human saliva are currently classed into two main categories, the high molecular weight MUC5B mucins and the low molecular weight MUC7 mucins. In our study, a sedimentation coefficient for mucin of ~4S was compared against a standard, Sigma bovine submaxilllary mucin (4S), which had a molecular weight of ~300 kDa (unpublished reports). This is in agreement with previous findings for MUC7 type mucins^35^, suggesting our 4S component represents the lower molecular weight MUC7 salivary mucin.

Secretory IgA (SIgA) is commonly found in saliva, and plays a critical role in mucosal immunity^36^. Is exists as a dimer, joined together by a J-protein domain and is known to have an s_20,w_ value of 11S and a molecular weight of 385 kDa^22,23^, which is consistent with our current findings. The levels of SIgA in our samples have been estimated at ~0.28 mg/mL, which is higher than some of the reported values^37^. Conversely, our results are in agreement with Aase, *et al*.^38^, who conducted a study to determine the SIgA concentrations from 30 healthy volunteers, measured by enzyme-linked immunosorbent assay (ELISA). Their analysis has found a concentration of 555.1 μg/mL and 435.7 μg/mL in sublingual and parotid secrtions respectively, which is higher than our reported values. Again, a question is raised on the viability of these reported values, as whole saliva samples usually undergo a centrifugation step before analysis, and this has been commonly found to result in loss of salivary proteins, either bound to aggregated mucus or bacteria^39^.

Based on our current hypothesis, the *in-vivo* partitioning of aroma compounds from green tea is key to the formation of characteristic notes during consumption i.e. isovaraldehyde (3,methyl,butanal), β-ionone, benzaldehyde. This has been attributed to α-amylase precipitation in the presence of EGCG. To validate this hypothesis, a GC-MS experiment was employed to measure the partitioning of aroma compounds from two model saliva systems, prepared in PBS 0.1 M (Supplementary Fig. 2). While both systems contained bovine submaxillary mucin at a 1 mg/mL, the second system also contained 1 mg/mL porcine pancreatic α-amylase. This design allowed us to identify the aroma compounds solely affected by the presence of α-amylase. As we hypothesized, the presence of α-amylase had a significant effect (*P* < 0.01) on the release of certain volatile aroma compounds (β-ionone, benzaldehyde and isovaraldehyde), which agrees with our data on human saliva showing compound specific interaction effects (Fig. 4).

This interaction has been studied previously by Lee, *et al*.^18^, using X-Ray crystallography. It was shown that the three hydroxyl groups of the epigallo and gallate groups form highly specific hydrogen bonds with the amino residues of α-amylase. Other studies have used fluorescence spectroscopy and UV-vis absorption spectroscopy to show that the interaction occurs near, or at the catalytic site of the enzyme^40^. Additionally, the degree of astringency has been reported to be dose-dependent on the number of galloyl rings present on the polyphenol chains^41,42^.

To conclude, the method presented in our study has proven to be a useful tool for testing *ex-vivo* interactions between human saliva and our model food system-green tea. To our knowledge, this study is the first investigation on the interactions of tea polyphenols and human saliva by analytical ultracentrifugation. Our analyses have allowed for a rapid means of identification of interacting species. Although other small molecular weight proteins identified within the saliva proteome may be involved in polyphenol binding, our results suggest that the characteristic flavour perception of green tea is largely influenced by the interaction of epigallocatechin gallate (EGCG) with salivary α-amylase. It was shown that the presence of EGCG leads to an increase in the relative viscosity of saliva by lowering amylase concentration, further adding a degree of complexity to our understanding of astringency as well as flavour release and perception.

It is well established that the addition of hydrocolloids to tea, i.e. milk, can lower the feeling of astringency by binding to tea flavonoids. Besides negating their beneficial effect, we have also established that their interactions with amylase is essential for the characteristic *in-vivo* flavour delivery, and this may be another reason why milk is not commonly added to green tea.

## Materials and Methods

### Saliva

“Stimulated” (SS) and “unstimulated” (US) saliva samples were kindly provided by Dr. Pavel Gershkovich and co-workers from the Centre for Biomolecular Sciences, University of Nottingham. All samples were collected in accordance with the ethical approval R12122013, Faculty of Medicine and Health Sciences Research Ethics Committee, Queens Medical Centre, Nottingham University Hospitals. Participation was voluntary and informed written consent was obtained. All data were held in accordance with the Data Protection Act.

Participants were asked to continually chew on a 5 cm × 5 cm piece of Parafilm. Samples were immediately flash frozen in liquid nitrogen and then stored at −80 °C until being defrosted for characterisation^5^. Loading and unloading of samples was carried out in a Level 2 microbiological safety cabinet. To account for the inter-individual variation, the sedimentation coefficient fingerprinting is based on a triplicate run for pooled saliva samples from five healthy volunteers. The samples have been centrifuged to 5000 rpm to remove the presence of large visible aggregates.

### Green tea

Twinings^TM^ Pure Green Tea (F09542) was purchased from the local supermarket. Samples were brewed according to the manufacturer, in a temperature controlled environment. The samples were mixed with saliva as the temperature of the brew cooled down to 60.0 °C. The GC-MS analysis was performed in triplicates. Milli-Q purified water was used throughout the sample preparation. Epigallocatechin gallate (PHR1333) was purchased from Sigma Ltd. (Poole,UK).

### Model saliva

The model saliva was prepared in 0.1 M phosphate buffered saline (PBS) according to Green^26^, using bovine submaxillary mucin (Type I-S) and α-amylase from porcine pancreas (Type VI-B), purchased from Sigma Aldrich (Dorset, UK). The final protein concentrations in the model saliva were adjusted to the concentrations of mucin and α-amylase in human saliva, neglecting the presence of SIgA and other proteins.

### Sedimentation Velocity-Analytical ultracentrifugation (SV-AUC)

Experiments were performed at 20.0 °C using the Optima XL-I analytical ultracentrifuge (Beckman, Palo Alto, USA) equipped with Rayleigh interference optics. Samples of 395 μL (and 405 μL solvent) were injected into the 12 mm double sector epoxy cells with sapphire windows and run at 40000 rpm (120 000 g). Scans were taken at 2 minutes intervals. The interference system produced data derived by recording changes in concentration (in fringe units) versus radial displacement. The results were analysed in SEDFIT using the least squares ls-g*(s) and the diffusion corrected c(s) processing methods^43^, by generating sedimentation coefficient distributions, s_20,w_ (in Svedberg units, S = 10^−13^ sec) normalised to standard conditions (viscosity, density of solvent at 20.0 °C)^44^.

### Differential Refractometry

The Atago DD-7 differential refractometer (Jencons Scientific, UK) was used to measure the concentrations of macromolecular solutions. A refractive index incremenet (*dn/dc*) values were 0.17 mL/g for mucin samples, 0.197 mL/g for α-amylase and 0.186 mL/g for SIgA, respectively^24^. Concentrations were monitored throughout the experimental analysis.

### Capillary viscometry

Flow times of solvent (t_0_) and solutions (t_s_) were measured using a semi-automated (Schott Geräte, Hofheim, Germany) U-tube Ostwald capillary viscometer immersed in a temperature controlled water bath at 20.00 °C. A constant volume of 2.0 mL was used for sampling. Physiological concentrations of saliva are sufficiently low (<1 mg/mL) to allow the assumption that no correction was needed for solution density, i.e. η_s_/η_0_ was assumed equal to t_s_/t_0_^45^. Therefore, to a reasonable approximation, the relative viscosity is equivalent to the dynamic viscosity^45^.

### Gas chromatography-mass spectrometry

The Trace 1300 series Gas Chromatograph coupled with the single-quadrupole mass spectrometer (Thermo Fisher Scientific, Hemel Hempstead, UK) was used. Samples were incubated at 55.0 °C for 20 minutes with intermittent stirring. Then, the solid phase microextraction (SPME) fiber (50/30 μm DVB/CAR/PDMS, Supelco, Sigma Aldrich, UK) was used to extract for 30 min then desorb for 1 min. Separation was carried out by a ZB-WAX capillary gas chromatography column (length 30 m, internal diameter 1 mm, 1.00 μm film thickness). The column temperature was held initially at 40.0 °C for 2 min, increased by 6.0 °C every minute up until 250.0 °C and held for 5 min. Full scan mode was chosen to measure volatile compounds (mass range from 20 to 300Da). A splitless mode was used, and the constant carrier pressure at 18 psi was applied. Volatiles were identified by comparison of each mass spectrum with either the spectra from authentic compounds analysed in our laboratory or with spectra in reference collections (the *NIST* Mass Spectral Library).

### Statistical analysis

The sedimentation coefficient fingerprint for human saliva and all GC-MS samples were analysed in triplicate in a randomised sample order and the analysis was made using analysis of variance (ANOVA) and Tukey’s post hoc test to identify significance (*P* < 0.05 and *P* < 0.01). All figures were made in Origin 7.5.

## Electronic supplementary material

Supplementary Material
