# Supplementary material for: Analytical ultracentrifugation in saliva research: Impact of green tea astringency and its significance on the in-vivo aroma release
Source: Sci Rep. 2018 Sep 6;8:13350. doi: 10.1038/s41598-018-31625-w (PMC6127339; doi:10.1038/s41598-018-31625-w)
Supplement: Supplementary file 1 — Supplementary Material [file 41598_2018_31625_MOESM1_ESM.pdf]

SUPPLEMENTARY

Analytical ultracentrifugation in saliva research: Impact of green tea astringency and its significance on the *in-vivo* aroma release.

Vlad Dinu[1,3], Chujiao Liu[3], Joseph Ali[4], Charfedinne Ayed[3], Pavel Gershkovich[4], Gary G. Adams[1,2], Stephen E. Harding[1] and Ian Fisk[3]*****.

[1]*National Centre of Macromolecular Hydrodynamics, Division of Food Sciences, School of Biosciences, University of Nottingham, Sutton Bonington Campus, Leicestershire, UK*

[2]*School of Health Sciences, Faculty of Medicine and Health Sciences, Queen's Medical Centre, Clifton Boulevard, Nottingham, UK*

[3] *Division of Food Sciences, School of Biosciences, University of Nottingham, Sutton Bonington Campus, Leicestershire, UK*

[4]*School of Pharmacy, University of Nottingham, Nottingham, United Kingdom, UK*

Corresponding author: ian.fisk@nottingham.ac.uk

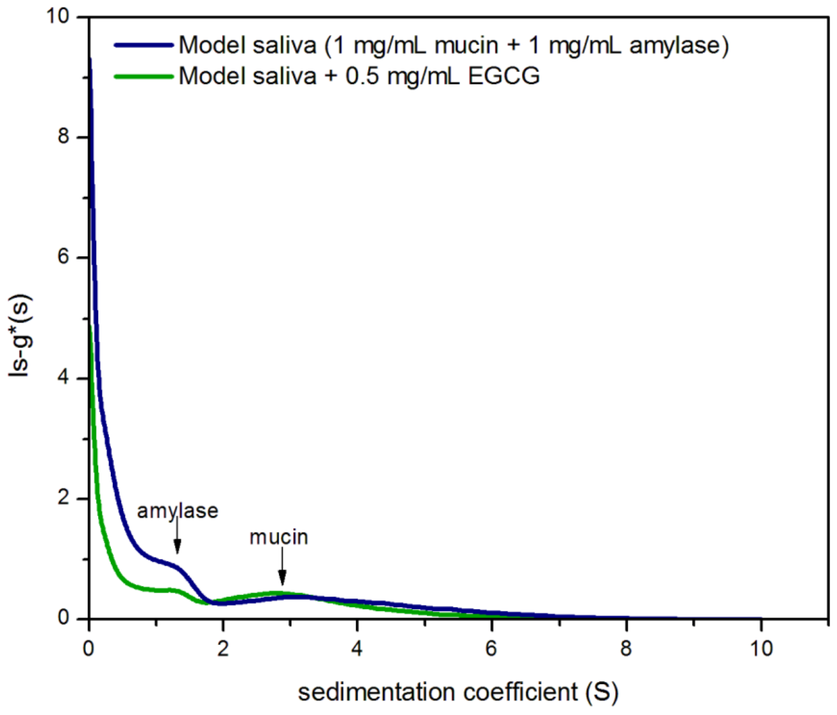

Supplementary figure 1. Sedimentation velocity, ls-g*(s) distributions for model saliva (blue) and what happens upon the addition of 0.5 mg/mL EGCG. The model saliva is made with 1 mg/mL bovine submaxillary mucin and 1 mg/mL porcine pancreatic α-amylase in 0.1 M PBS, pH 6.8. The distributions are broad and show two components: α-amylase ranging from 0 to 2S and mucin ranging from 2 to 8S. The addition of 0.5 mg/mL EGCG results in a decrease in the α-amylase peak, equivalent to 47%, as indicated by the area under the curve for the first peak (Area = (X1+X1)/2*(S2-S1), where X represents data points for the sedimentation coefficient distribution curve and S represents the data points range within the sedimentation coefficient distribution).

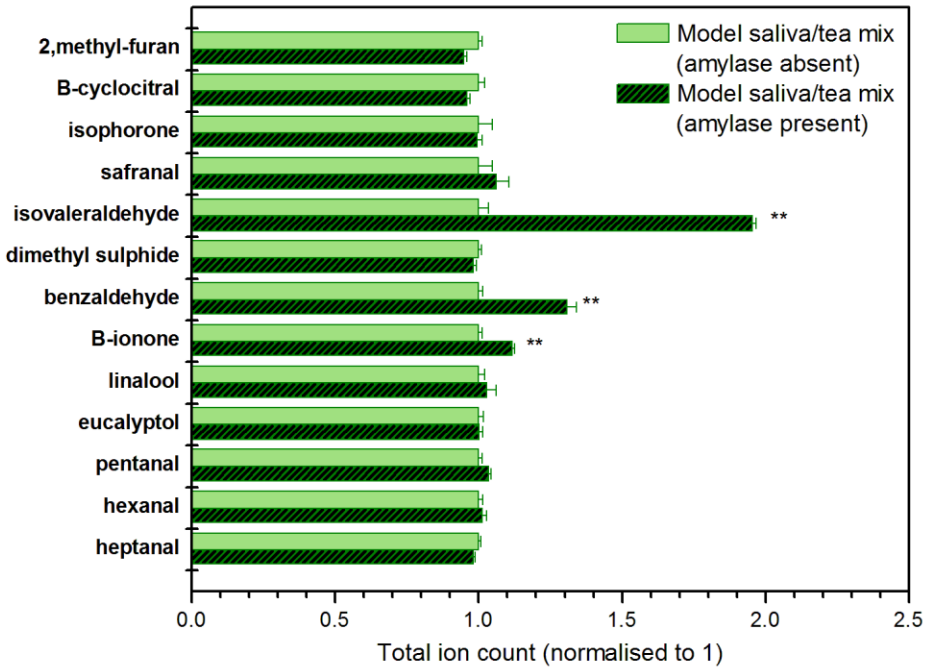

Supplementary figure 2. Effect of green tea on the release of aroma compounds from model saliva in the presence (dashed green) and absence (green) of α-amylase. The experiment was made using bovine submaxillary mucin at 1 mg/mL and 1 mg/mL porcine pancreatic α-amylase. Each model saliva sample was mixed with green tea brew at a 1:1 ratio. The comparison is made by Tukey's post hoc test to calculate the P-values (P<0.01**). The data shown as mean +/- SD, n=3.
